# Case Report: Carcinoma Ex‐Pleomorphic Adenoma With Concurrent Submandibular Gland Lithiasis—A Rare Association With Potential Pathophysiological Implications

**DOI:** 10.1002/ccr3.71327

**Published:** 2025-10-30

**Authors:** Huiquan Lou, Yichao Xia, Shengjie Shao, Xiang Liu, Li Deng, Hongbin Yu, Zengzheng Li

**Affiliations:** ^1^ Oral and Maxillofacial Surgery Department The First People's Hospital of Yunnan Province/The Affiliated Hospital of Kunming University of Science and Technology Kunming Yunnan China; ^2^ Pathology Department The First People's Hospital of Yunnan Province/The Affiliated Hospital of Kunming University of Science and Technology Kunming Yunnan China; ^3^ Department of Stomatology Yan'an Hospital of Kunming City, The Affiliated Yan'an Hospital of Kunming Medical University Kunming Yunnan China; ^4^ Department of Hematology The First People's Hospital of Yunnan Province/The Affiliated Hospital of Kunming University of Science and Technology Kunming Yunnan China

**Keywords:** carcinoma ex‐pleomorphic adenoma, chronic inflammation, diagnostic algorithm, malignant transformation, submandibular gland lithiasis

## Abstract

This case report details the exceptionally rare co‐occurrence of submandibular gland lithiasis and carcinoma ex‐pleomorphic adenoma (CXPA), representing only the fourth documented instance worldwide. The patient, a 79‐year‐old male with a 10‐year history of recurrent submandibular symptoms, was found to have both ductal and intraglandular stones alongside non‐uniform glandular enlargement. Post‐surgical analysis confirmed CXPA. The patient remained disease‐free with good functional outcomes at 24‐month follow‐up. This case provides valuable insights, suggesting a potential pathophysiological link between the chronic inflammation associated with long‐standing lithiasis and the malignant transformation leading to CXPA. It underscores the importance of comprehensive diagnostic evaluation for chronic or atypical submandibular gland conditions to rule out concurrent malignancy.

**Summary**

- Concurrent submandibular lithiasis and carcinoma ex‐pleomorphic adenoma, though exceptionally rare, suggest chronic inflammation from longstanding salivary stones may promote malignant transformation.
- In patients with chronic or atypical submandibular lithiasis, comprehensive.

## 1 Introduction

Sialolithiasis represents one of the most common pathological conditions affecting salivary glands, second only to mumps. The condition exhibits a male predominance with a 2:1 ratio compared to females [1]. Submandibular glands are most frequently affected (83%), followed by parotid (10%) and sublingual glands (7%) [2]. This distribution pattern is attributed to several factors including alkaline pH, calcium content, viscosity, decreased saliva flow rate, and anatomical characteristics specific to the submandibular gland system. The diameter of salivary lithiasis typically ranges from a few millimeters to several centimeters, with most measuring under 10 mm. Lithiasis exceeding 15 mm in diameter, classified as “giant lithiasis,” represents a relatively uncommon finding in clinical practice [3].

Carcinoma ex‐pleomorphic adenoma (CXPA) represents the predominant form of malignant transformation of pleomorphic adenoma (PA) in salivary glands [4]. According to the World Health Organization classification, malignant transformations of pleomorphic adenoma encompass three distinct categories: CXPA, carcinosarcoma, and metastasizing PA, with CXPA being the most prevalent. CXPA accounts for approximately 3.6% of all salivary gland tumors and constitutes between 5% and 15% of all malignant salivary gland neoplasms [5, 6].

A systematic review of the literature reveals the extreme rarity of concomitant submandibular gland lithiasis and CXPA [7]. Our comprehensive search identified only three previously documented cases worldwide, making this the fourth reported instance of this association [8, 9, 10]. This scarcity raises intriguing questions regarding the potential relationship between these two pathological entities, particularly considering the accumulating evidence suggesting that chronic inflammation may play a significant role in the malignant transformation of pleomorphic adenomas [6, 7].

Recent molecular studies have begun to elucidate the potential mechanisms through which chronic inflammation might influence neoplastic transformation [11, 12, 13]. Inflammatory mediators, including cytokines, chemokines, and growth factors, create a microenvironment that can promote genetic instability, cellular proliferation, and inhibition of apoptosis [6]. In the context of salivary glands, repeated cycles of inflammation and repair associated with lithiasis could potentially create conditions conducive to the malignant transformation of pre‐existing pleomorphic adenomas through mechanisms involving oxidative stress, DNA damage, and alterations in cellular signaling pathways [4, 14].

Understanding the potential association between these conditions is of significant clinical importance as it may influence diagnostic approaches and management strategies for patients presenting with submandibular lithiasis, particularly those with atypical features or prolonged symptomatology. This case report aims to contribute to the limited body of knowledge regarding this rare association and to propose potential pathophysiological mechanisms that might underlie the co‐occurrence of these distinct entities.

## 2 Case History

**FIGURE 1:**
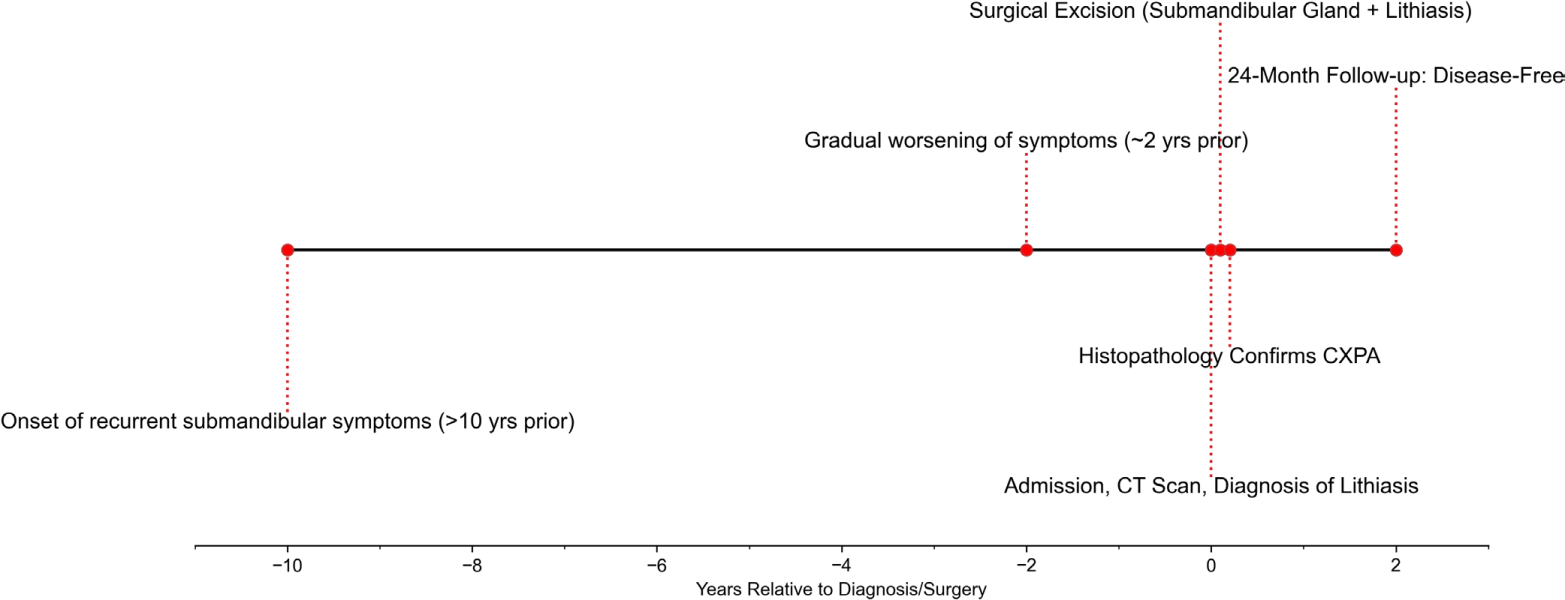
Clinical timeline of the patient. The timeline illustrates key events from the onset of symptoms over 10 years prior to diagnosis, through worsening symptoms, admission, diagnostic imaging (CT), surgical intervention, histopathological confirmation of carcinoma ex‐pleomorphic adenoma (CXPA) and lithiasis, to the 24‐month disease‐free follow‐up period. Time is shown in years relative to the point of admission and initial diagnosis/surgery (Year 0).

A 79‐year‐old male patient was admitted to our institution with a diagnosis of right submandibular gland lithiasis based on a history of recurrent swelling and pain in the right submandibular region persisting for more than 10 years. The patient reported intermittent episodes of pain exacerbation, particularly during meals, with gradual worsening of symptoms over the past two years. The patient's medical history was significant for hypertension of over 20 years duration, which was effectively managed with antihypertensive medication. He denied any history of radiation exposure, familial salivary gland disorders, or autoimmune conditions. The patient presented with a complex history spanning over a decade. The key events in the patient's clinical course, from initial symptom onset to the most recent follow‐up, are summarized in Figure 1.

## 3 Differential Diagnosis, Investigations and Treatment

Physical examination revealed notable bilateral facial asymmetry with pronounced swelling and fullness in the right submandibular region compared to the left side. Bimanual palpation demonstrated an enlarged, firm, and tender right submandibular gland. Additionally, firm nodules were palpable along the right floor of the oral cavity, which were consistent with ductal lithiasis. Salivary stimulation produced minimal saliva from the right Wharton's duct compared to normal flow from the left side, suggesting significant functional impairment.

**FIGURE 2:**
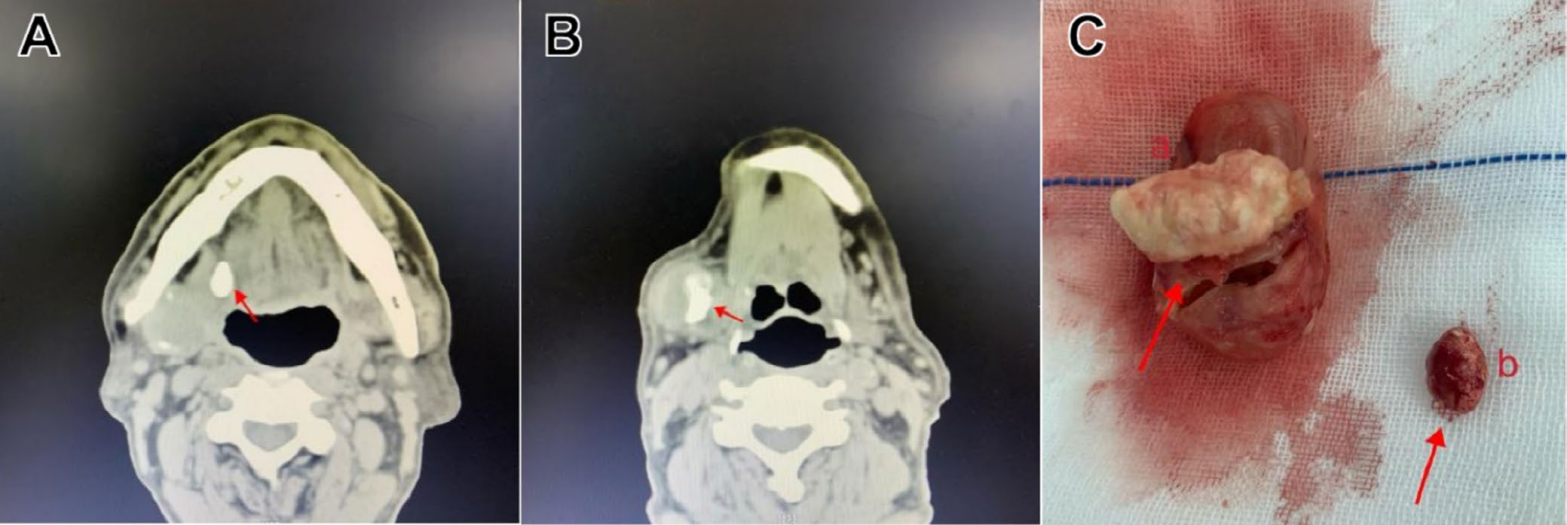
Location of the patient's lithiasis. Preoperative maxillofacial CT examination: (A) Lithiasis in the submandibular gland duct; (B) Lithiasis in the submandibular gland; (C) Lithiasis removed from the submandibular gland and lithiasis removed from the submandibular gland duct. The red arrow indicates the location of the lithiasis.

Given the chronic nature of the condition, the multidisciplinary team decided to proceed with advanced imaging studies to fully characterize the extent of pathology. Computed tomography (CT) examination revealed nodular high‐density imaging at the right tongue base, with the largest lithiasis measuring approximately 13 mm × 8 mm (Figure 2A). The right submandibular gland exhibited non‐uniform volumetric enlargement (34 mm × 32 mm) with irregular density. Several nodular high‐density structures were identified both within the glandular parenchyma and along its periphery, with one particularly prominent lithiasis measuring approximately 17 mm × 7 mm (Figure 2B).

These imaging findings, combined with the patient's protracted clinical history, raised concerns beyond simple lithiasis. Although chronic sclerosing sialadenitis was considered the most likely diagnosis, the differential diagnosis included potential neoplastic processes given the non‐uniform enlargement pattern and the patient's advanced age. The multidisciplinary team extensively discussed the management approach, considering both the need for symptom relief and the importance of a definitive diagnosis.

After a comprehensive preoperative evaluation to assess anesthesia and surgical risks, the decision was made to proceed with complete surgical excision of the right submandibular gland and removal of all identifiable lithiasis under general anesthesia. This approach was selected over more conservative measures such as sial endoscopy given the chronic nature of the condition, the presence of both ductal and intraglandular lithiasis, and the need to exclude potential malignancy. Intraoperatively, larger lithiasis structures were identified within the submandibular gland parenchyma, while smaller calculi were located within the submandibular duct system (Figure 2C).

## 4 Conclusion and Results (Outcome and Follow‐Up)

**FIGURE 3:**
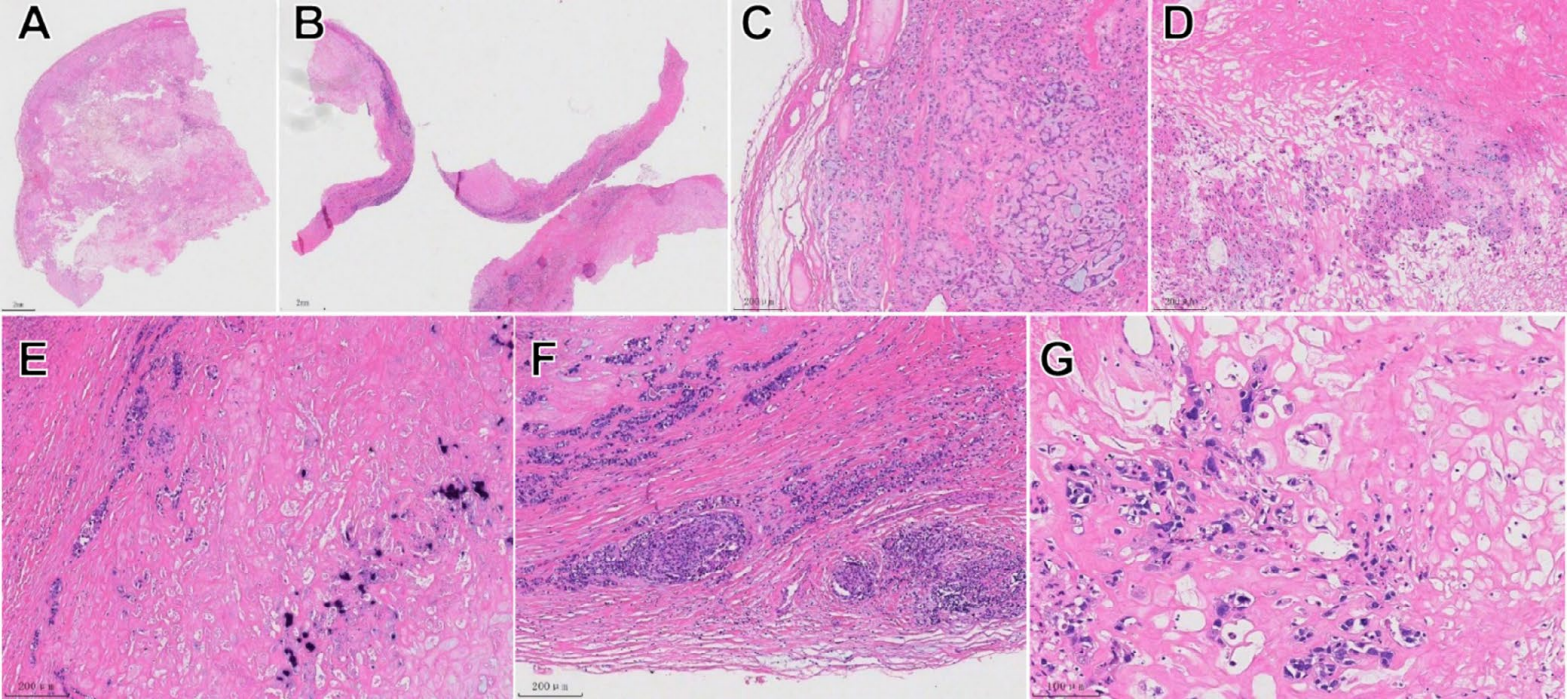
Histopathological examination (H&E staining). (A, B) Low magnification shows the intact tumor capsule, cystic solidity, and notable degeneration and sclerosis. (C) H&E ×100, showing the intact tumor envelope composed of glandular epithelium, myoepithelium, and chondroid matrix. (D) H&E ×100, showing hardening. (E) H&E ×100, showing degeneration and calcification. (F) H&E ×100, showing a strip‐like, nested, and glandular arrangement of nuclear hyperchromatic cells near the capsule, with visible nerve invasion. (G) H&E ×200, showing enlarged, hyperchromatic, and atypical cells in the central area of the tumor, arranged in a disordered manner.

Postoperative histopathological examination revealed unexpected findings beyond the anticipated chronic sialadenitis. H&E staining demonstrated an intact tumor capsule with both cystic and solid structural characteristics, showing areas of degeneration and calcification. The tumor exhibited a complex composition of glandular epithelium, myoepithelium, and chondroid matrix. Concerning features included the presence of strands, nests, and glandular arrangements of deeply stained nuclear cells near the tumor capsule, with visible nerve invasion. The central tumor region contained enlarged, deeply stained nuclei and atypical cells arranged in a disordered pattern (Figure 3A–G).

**FIGURE 4:**
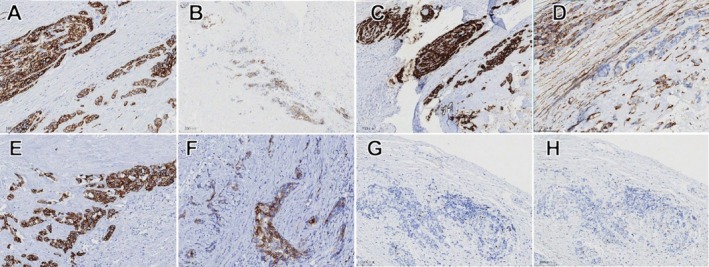
Immunohistochemistry. (A) Positive expression of PCK in cancer cells. (B) Positive expression of LCK in cancer cells. (C) Positive expression of CK7 in cancer cells. (D) Positive expression of CD117 in cancer cells. (E) Positive expression of CK5/6 in cancer cells. (F) Positive expression of SMA in some cancer cells. (G) Negative expression of P40 in cancer cells, with slight positive expression in myoepithelium. (H) Negative expression of P63 in some cancer cells, with a few myoepithelial cells showing positive expression.

Comprehensive immunohistochemical analysis was performed to further characterize the neoplasm. Results demonstrated positive expression of PCK, LCK, CK7, CD117, and CK5/6 (Figure 4A–E). Additionally, some cells exhibited positive expression of SMA, while a smaller subset of myoepithelial cells showed positive staining for P40 and P63 (Figure 4F–H). These immunohistochemical findings, combined with the histopathological features, were consistent with a diagnosis of carcinoma ex‐pleomorphic adenoma.

Based on these results, the patient was diagnosed with right submandibular gland lithiasis concurrent with Carcinoma ex‐pleomorphic adenoma. The case was presented at a multidisciplinary tumor board to determine the need for adjuvant therapy. Given the patient's advanced age, the complete surgical excision with clear margins, the absence of detectable cervical lymph node involvement, and the patient's preference, the consensus decision was to proceed with careful surveillance without additional treatment. The patient was enrolled in a structured follow‐up program with regular clinical evaluations, imaging studies, and quality‐of‐life assessments.

This report details the fourth documented case of concurrent submandibular gland lithiasis and CXPA, a rare but clinically significant association. Our findings support the hypothesis that chronic inflammation from long‐standing lithiasis may act as a promoter for malignant transformation in a pre‐existing pleomorphic adenoma. This case underscores the critical need for a high index of suspicion and comprehensive diagnostic evaluation for patients presenting with chronic or atypical submandibular gland conditions to ensure timely detection of potential underlying malignancies. It also provides a basis for future research into the intricate relationship between chronic inflammation and salivary gland carcinogenesis.

Postoperative histopathological examination unexpectedly diagnosed a rare case of carcinoma ex‐pleomorphic adenoma (CXPA) concurrent with submandibular lithiasis, confirmed by histological findings of nerve invasion and atypical cells, and positive immunohistochemical staining for markers including PCK, CK7, and CD117. Given the patient's advanced age and complete surgical excision with clear margins, a multidisciplinary team opted for close surveillance without adjuvant therapy, resulting in the patient remaining disease‐free with good functional outcomes at 24‐month follow‐up. As the fourth documented case globally, this report supports the hypothesis that chronic inflammation from long‐standing lithiasis may act as a promoter for malignant transformation, highlighting the need for a high index of suspicion in chronic or atypical submandibular gland conditions.

## 5 Discussion

This case presents a rare and clinically significant association between submandibular gland lithiasis and carcinoma ex‐pleomorphic adenoma (CXPA). While both conditions individually are well documented in medical literature, their concurrent presentation is exceptionally uncommon. Our comprehensive literature review identified only three previously reported cases worldwide, establishing this as the fourth documented instance of this unique association.

### 5.1 Epidemiological and Clinical Considerations

Salivary gland lithiasis predominantly affects the submandibular gland, accounting for approximately 80% of all cases [15]. The predilection for this particular gland is attributed to multiple factors, including the relatively alkaline pH of submandibular saliva, its higher calcium and mucin content, the tortuous course of Wharton's duct, and gravitational considerations affecting saliva flow [16]. Our patient's presentation aligned with classic sialolithiasis symptoms, including recurrent post‐prandial swelling and pain, though the 10‐year symptomatic period exceeds the typical duration reported in most cases. The prolonged clinical course in our patient raises important diagnostic considerations. Chronic sialadenitis secondary to lithiasis typically produces fibrotic changes that can obscure concurrent pathologies, potentially delaying the diagnosis of co‐existing neoplasms [15]. This underscores the importance of comprehensive diagnostic evaluation, including advanced imaging, in cases with atypical features or extended symptomatology [6]. In comparing our case with the three previously reported instances of concurrent submandibular lithiasis and CXPA, several distinctive features emerge [8, 9, 10]. Our patient exhibited a longer symptomatic period prior to diagnosis (10+ years vs. 2–5 years in previous reports), larger lithiasis (17 mm vs. 5–12 mm in reported cases), and a unique immunohistochemical profile, particularly regarding CD117 expression, which was absent in two of the three previously reported cases [8, 9, 10]. These differences may reflect variations in the underlying pathophysiological processes and highlight the heterogeneity of this rare association.

### 5.2 Pathophysiological Hypothesis

The co‐occurrence of lithiasis and CXPA in our patient raises intriguing questions regarding potential pathophysiological relationships between these distinct entities. We propose a novel hypothesis that chronic inflammation associated with long‐standing lithiasis may contribute to or accelerate the malignant transformation of pre‐existing pleomorphic adenoma through several potential mechanisms. Persistent inflammation creates a cytokine‐rich microenvironment that can promote genomic instability and cellular proliferation [17]. Recent studies have demonstrated elevated levels of pro‐inflammatory mediators including IL‐1β, IL‐6, TNF‐α, and COX‐2 in salivary tissues affected by chronic sialadenitis [6]. For instance, the generation of reactive oxygen species (ROS) during chronic inflammation is known to induce DNA damage and epigenetic alterations [18, 19], Such molecular damage could potentially trigger the malignant transformation of pre‐existing pleomorphic adenoma cells. Inflammatory processes have been shown to promote EMT, a critical step in malignant progression [20]. The expression pattern of cytokeratins and SMA in our case supports the occurrence of EMT within the tumor. Inflammatory signaling can activate tissue stem/progenitor cells, which may increase susceptibility to oncogenic transformation [12]. The positive expression of CD117 (c‐Kit) in our case is particularly relevant in this context, as this marker is associated with stem cell properties and has been implicated in the pathogenesis of various malignancies [21]. The immunohistochemical profile observed in our patient provides circumstantial support for this hypothesis. The positive expression of CD117, coupled with the specific pattern of cytokeratin expression, suggests the activation of cellular pathways that have been associated with inflammation‐driven carcinogenesis in other organ systems [17]. While this hypothesis requires further validation through molecular and experimental studies, it provides a plausible framework for understanding the potential relationship between these co‐occurring pathologies.

### 5.3 Diagnostic Challenges and Management Considerations

The concurrent presentation of lithiasis and CXPA presents significant diagnostic challenges. In our case, the chronic sclerosing sialadenitis resulting from long‐standing lithiasis complicated the clinical assessment of potential neoplastic processes. Palpation alone proved insufficient for detecting the underlying CXPA, highlighting the importance of advanced imaging in the evaluation of chronic submandibular pathologies. CT imaging played a crucial role in our diagnostic process [22], demonstrating not only the lithiasis but also revealing subtle features suggestive of potential neoplastic changes, including non‐uniform glandular enlargement and irregular density patterns [3]. These findings prompted the decision for complete surgical excision rather than more conservative approaches [23]. Based on our experience and review of the literature, we propose a modified diagnostic algorithm for evaluating chronic submandibular pathologies that incorporates careful consideration of potential concurrent neoplastic processes, particularly in patients with: Prolonged symptomatology (> 5 years), Non‐uniform glandular enlargement, Irregular density patterns on imaging, Incomplete resolution of symptoms between acute episodes, or Age > 50 years. The therapeutic approach for concurrent lithiasis and CXPA must address both pathologies while prioritizing oncological principles. Complete surgical excision, as performed in our case, represents the treatment of choice [24], allowing for both symptomatic relief and comprehensive histopathological evaluation. The decision regarding adjuvant therapy should be individualized based on histopathological features, marginal status, and patient‐specific factors [25]. In our case, given the complete excision, clear margins, absence of lymph node involvement, and the patient's preference, surveillance without adjuvant therapy was selected. Long‐term follow‐up is essential for monitoring potential recurrence or metastatic spread. Our patient has remained disease‐free for 24 months post‐surgery, with excellent functional outcomes and quality‐of‐life scores as assessed using standardized measurement tools. This favorable outcome aligns with previous reports, suggesting that early and complete excision of these lesions is associated with good prognosis despite the typically aggressive nature of CXPA.

## Author Contributions

**Huiquan Lou:** validation, visualization, writing – original draft. **Yichao Xia:** resources, software. **Shengjie Shao:** methodology, supervision. **Xiang Liu:** conceptualization, resources. **Li Deng:** software, supervision. **Hongbin Yu:** methodology, project administration. **Zengzheng Li:** conceptualization, funding acquisition, methodology.

## Ethics Statement

The study involving a human participant was reviewed and approved by the Ethics Committee of Yunnan Provincial First Hospital (Approval No. 2020KYS011).

## Consent

Written informed consent was obtained from the patient for publication of this case report and any accompanying images. A copy of the written consent is available for review by the Editor‐in‐Chief upon request.

## Conflicts of Interest

The authors declare no conflicts of interest.

## Data Availability

The original contributions presented in the study are included in the article/Supporting Information, further inquiries can be directed to the corresponding author/s.
